# A 10‐Year Longitudinal Study of Muscle Morphology and Performance in Masters Sprinters

**DOI:** 10.1002/jcsm.13822

**Published:** 2025-04-27

**Authors:** P. W. Hendrickse, B. Hutz, M. T. Korhonen, H. Degens

**Affiliations:** ^1^ Lancaster Medical School Lancaster University Lancaster UK; ^2^ Faculty of Sport and Health Sciences University of Jyväskylä Jyväskylä Finland; ^3^ Department of Life Sciences, Institute of Sport Manchester Metropolitan University Manchester UK; ^4^ Institute of Sport Science and Innovations Lithuanian Sports University Kaunas Lithuania

**Keywords:** ageing, atrophy, capillarisation, histology, masters athletes, muscle biopsy, MVC, performance, skeletal muscle, sprinters

## Abstract

**Background:**

Both longitudinal and cross‐sectional studies have demonstrated that muscle mass, strength and power are lost with ageing. Although longitudinal studies have shown changes in muscle morphology and function in sedentary, healthy active and endurance‐trained older people, less is known about such age‐related changes in sprint athletes. It has been proposed that active older people may provide a better study of healthy ageing not confounded by factors of inactivity and other unhealthy behaviours. Given that the training regimens of masters sprinters consist of strength and sprint training that elicit gains in muscle force, power and mass, sprinters may not suffer from measurable decrements in muscle strength, functional performance or morphology over a 10‐year period.

**Methods:**

To investigate this, *m. vastus lateralis* (VL) biopsies were taken from 24 masters sprinters aged 48–85 years at baseline and 10 years later. Immunofluorescent staining of slides taken from these biopsies was used to assess fibre type composition, fibre cross‐sectional area (FCSA) and capillarisation. In addition, VL thickness was assessed using B‐mode ultrasonography, maximum voluntary contraction (MVC) of knee extension was measured with an electromechanical dynamometer, and the flight time of a counter movement jump was determined with a contact matt. 60‐m sprint times were measured using double‐beam photocell gates connected to an electronic timer.

**Results:**

FCSA, fibre‐type composition, capillarisation and VL thickness had not changed significantly after 10 years. The decrease in jump power (−9.5% ± 5.7, *p* < 0.001) was attributable to a concomitant decrease in knee extension MVC (−21.0% ± 20.4, *p* < 0.001), not slowing of the muscle. Athletes demonstrated reduced 60‐m sprint performance after 10 years (+14.2% increase in sprint time ± 12.4, *p* < 0.001) with greater loss in performance found in older participants (stepwise regression *p* < 0.004). Similarly, the loss of jump power found in the follow‐up measurement (−9.47% ± 5.7, *p* < 0.001) was larger in the older participants (stepwise regression *p* < 0.001). However, no changes in muscle function or performance were significantly related to years of training or training volume.

**Conclusions:**

Masters sprinters aged 48–85 maintained muscle histological characteristics over 10 years, but their training was unable to offset decrements in sprint performance and power that were attributable to a loss in force generating capacity, but not slowing of the muscle.

## 1 Introduction

Performance in power‐based sport declines during ageing, with accelerated reductions after the age of 70 years [1]. This is demonstrated by the accelerated increase in masters world record times for the 100‐m sprint when plotted by age group [2] and the performance of other track and field athletes [1].

Ageing‐related decrements in muscle mass [3, 4], muscle strength [5] and function [6, 7, 8, 9, 10] are well documented, and consequent mobility limitations are predictive of poor health and quality of life [11]. It has been suggested that much of the deterioration found in muscle with ageing is attributable to physical inactivity, and longitudinal studies of active older people, such as masters athletes, may allow the study of healthy ageing not confounded by the impact of decreased levels of physical activity and other unhealthy behaviours [12, 13].

It has been shown that resistance training in older people can lead to capillary proliferation [14] and that masters athletes demonstrate a greater capillary‐to‐fibre ratio (C:F) than young athletes [15]. Therefore, we predict that the microvasculature will be maintained in masters sprinters over a 10‐year period.

The potential significance of physical activity, even for the function of individual muscle fibres, is illustrated by the reported modulation of impaired single fibre contractile function with age by the level of physical activity of an individual [16] and adds to the benefit of greater levels of physical activity being associated with a larger muscle mass in old age [10]. Previous studies of muscle fibre characteristics in ageing athletes have largely focussed on endurance‐trained populations and have, for the most part, found benefits to greater activity. For example, Pollock et al. conducted a cross‐sectional study of highly active cyclists aged 55–79 and found no associations between muscle fibre cross‐sectional area (FCSA) and age [13]. Similarly, a 20‐year follow‐up study of long distance runners found no reductions in muscle fibre area, except for those who continued to train for elite competition [17], suggesting that substantial endurance training volume may be detrimental to the maintenance of muscle mass in older people. Interestingly, a longitudinal study of sedentary older men found that muscle fibre size was maintained over a 12‐year period, although computerised tomography showed reductions in the cross‐sectional area of all thigh muscles [8]. Given that resistance training, as used by sprinters [18, 19], is effective in increasing muscle mass, fibre size and strength [20, 21], one might expect that such a preservation of muscle mass, fibre size and strength will be evident in masters athletes who include regular resistance exercise in their training programme as is the case for sprinters. Yet, to our knowledge, this has not yet been explored in a longitudinal study in masters power or sprint athletes.

Previous studies of this cohort have found promising observations for musculoskeletal health. Although cross‐sectional observations of muscle have shown greater occurrence of motor neuron loss‐induced fibre type grouping with advancing age [22], this phenomenon did not occur over a 10‐year period in these participants [23]. In addition, a study of bone structural, strength and densitometric parameters found that tibial properties were maintained or improved in this group of middle‐aged and older male sprint athletes [18], in contrast to cross‐sectional studies showing an age‐related decline in athletes [24].

Despite a maintained muscle morphology during ageing, endurance athletes did show a significant reduction in maximal oxygen uptake [13, 17, 25]. Likewise, the age‐related reduction in sprint performance [2, 26, 27] may be disconnected from age‐related changes in muscle morphology, and indeed, it has been suggested, primarily based on observations in endurance athletes, that there are different rates of decline in performance and muscle morphology [12]. To investigate this in sprint athletes, we have taken performance measures, whole muscle thickness and histological analyses from masters sprinters before and after a 10‐year period. The objectives of this longitudinal study of performance and muscle morphology are to (i) elucidate morphological and/or physiological changes that occur during this period, (ii) measure strength, jump and sprint performance before and after 10 years and (iii) determine whether self‐reported training age and/or training volume affects morphology and/or performance.

Based on previous cross‐sectional and longitudinal studies of older athletes and physically active people, we hypothesise that in age 48–85 sprint athletes, (1) muscle thickness and muscle FCSA and (2) capillarisation will be maintained but that (3) an ageing‐related reduction in strength, sprint and jump performance will be seen.

## 2 Methods

### 2.1 Participants

Twenty‐four 48‐ to 85‐year‐old male masters sprinters were recruited by personal letters to members of Finnish track and field organisations. To be included in the study, the athletes had to have a long‐term sprint training background and success in international or national championships in 100‐, 200‐ and 400‐m sprint events. Participants were deemed healthy by reference to their detailed medical histories, and those over 55 years were further evaluated for clinical evidence of cardiovascular diseases by a focussed medical examination based on resting electrocardiograms and blood pressure measurements [28]. Written consent was obtained from all subjects. The Ethics Committee of the University of Jyväskylä, and the Ethics Committee of the Central Finland Health Care District, in conformity with the Declaration of Helsinki, approved the study.

### 2.2 Anthropometry, Muscle Thickness and Biopsies

Participant anthropometry was determined, and muscle thickness of the *vastus lateralis* muscle (VL) was measured at the mid‐region of the muscle using B‐mode ultrasonography with a 5‐cm linear‐array probe (7.5 MHz) and was determined as the distance from the adipose tissue–muscle interface to the intermuscular interface at 50% of the distance between the lateral condyle of the femur and the greater trochanter. Muscle biopsies were taken using a needle biopsy technique at the same location as the ultrasound measurement. Before the biopsy, the area was cleaned with an antiseptic solution and then anaesthetised with 1% lidocaine containing epinephrine. A 5‐mm needle was inserted into the muscle belly at a depth of 1.5–2.5 cm below the surface of the skin, and 100–150 mg of muscle tissue was removed with suction. Care was taken to achieve a consistent biopsy depth because of potential variation in fibre‐type distribution and size from the superficial to deep vastus lateralis. One piece of this biopsy was prepared for cryosection in optimal cutting temperature (OCT) embedding media [28].

### 2.3 Sprint Performance and Functional Measurements

All 24 participants performed standing‐start 60‐m sprint trials (two maximum‐effort attempts) after a warmup and an average of the two attempts was used in the analysis. Maximal isometric voluntary contraction (MVC) of the knee extensor muscles was measured in bilaterally using a dynamometer custom‐made at the University of Jyväskylä at 107° knee angle and 110° hip angle (two practice contractions followed by three to four maximum‐effort trials, each interspersed with 1–1.5 min rest. The best maximum value was used for further analysis. Vertical counter movement jumps were performed to determine muscle velocity and power using a contact mat (Newtest, Oulu, Finland) that recorded the flight time of the vertical jump. Contact‐mat‐derived measures have been shown to be comparable to those from the gold standard method, the force platform [29]. One practice followed by three to four maximum‐effort attempts, each interspersed with 1–1.5 min rest). The height of rise of the body's centre of gravity was calculated from the flight time. The highest jump with an acceptable technique was used for the analyses.

We plotted take‐off velocity vs. body mass (BM) to MVC ratio (BM/MVC) where BM/MVC can be seen as P/Po in the force–velocity relationship [30, 31]. Body mass adjusted to MVC gives an indication of maximal force needed to lift the body mass. Therefore, if the velocity at a given BM/MVC after 10 years is unchanged, it indicates no change in the contractile properties of the muscle.

### 2.4 Morphometry

In a previous study, we validated that the long‐term storage of the first biopsies did not affect the quality of the immunofluorescent staining [32]. Sections were fixed with methanol for 10 min at −20°C and then washed for 5 min in phosphate‐buffered saline (PBS). After 10‐min permeabilisation with 0.2% Tween 20 (Sigma‐Aldrich) in PBS and a further 5‐min wash in PBS, sections were incubated in blocking solution (10% normal goat serum (Vector Labs) in PBS) for 1 h. The sections were then incubated in a primary antibody cocktail in blocking solution for 1 h containing BA‐D5 (1:40, Developmental Studies Hybridoma Bank, University of Iowa), BF‐35 (1:40 Developmental Studies Hybridoma Bank, University of Iowa) and anti‐dystrophin (1:200, Abcam, ab125277). Slides were then washed 3 × 5 min in PBS before incubation with a secondary antibody cocktail in blocking solution containing AlexaFluor 405 goat anti‐mouse IgG2b (1:500, against BA‐D5, Jackson ImmunoResearch), AlexaFluor488 goat anti‐mouse IgG1 (1:500, against BF‐35, Thermo Fisher Scientific), AlexaFluor564 Goat anti‐rabbit IgG (1:500, against anti‐dystrophin, Thermo Fisher Scientific) and *Ulex europaeus* Agglutinin I lectin (1:200, UEA1, DyLight 649, Vector Labs). After further 3 × 5‐min washes in PBS, slides were mounted with ProLong Diamond (Thermo Fisher Scientific). A Zeiss LSM 700 confocal microscope was used to obtain microscopic images were with a 10× objective. Fibres were classified as Type I fibre if they stained positively for BA‐D5 as Type IIa with a positive stain for BF‐35, but not BA‐D5, and Type IIx fibres showed no staining with either antibody. Capillaries were manually identified by positive staining for *U. europaeus* Agglutinin I lectin. During image analysis, the rater was blinded to the age of the participants. Images were analysed with DTect software (https://ora.ox.ac.uk/objects/uuid:6d128833‐4c00‐46bd‐b7aa‐10145e5091b9) [33]. Fibre outlines were determined semi‐automatically using thresholding and manual removal of non‐specific staining. Capillaries were manually identified. Using these data, FCSA, fibre type, capillary density (CD in mm^−2^) and C:F were determined. Capillary locations and fibre data were used to calculate capillary domains; areas of tissue surrounding each capillary delineated by equidistant boundaries from neighbouring capillaries. These domains were used to determine the local capillary‐to‐fibre ratio (LCFR: the sum of the fractions of each domain which overlap the fibre), capillary fibre density (CFD: LCFR divided by the FCSA) and the logarithmic standard deviation of the radius of the capillary domains (Log_R_SD), an index of the heterogeneity of capillary spacing. A minimum of 80 fibres were analysed per sample. These participants are a subset of those studied by Messa et al. [23] and Suominen et al. [18]. In contrast to Messa et al. [23] where ATPase staining was used to determine Type I and II fibre types, here we classified fibres through immunofluorescent staining as Type I, Type IIa and Type IIx fibre types and also analysed capillarisation. These parameters were related to maximum isometric voluntary contraction (iMVC) and countermovement jump (CMJ) performance, in addition to the sprint performance included by Messa et al. [23] to indicate the high‐performance status of the study group.

### 2.5 Statistics

Assuming an alpha of 0.05 and power of 0.80, to observe a reduction in knee extension torque of 19.3% (±7.7) [34], an a priori calculation using G*Power determined that a minimum sample size of 5 was required [35].

All variables were examined for normality using Shapiro–Wilk tests. Two‐tailed paired *t*‐tests or Wilcoxon matched pairs signed‐rank tests were used to detect significant differences between baseline and 10‐year follow‐up in normally and non‐normally distributed data sets, respectively. If a significant difference was found, a stepwise regression of the change in the measure was used to determine if there was a relationship with age, years of training and/or training hours/week. These analyses were performed with IBM SPSS version 29.

Simple linear regressions were used to determine the cross‐sectional relationship between age and each dependent variable separately at baseline and at 10‐year follow‐up. Differences between the slopes of baseline and 10‐year follow‐up were determined with an extra sum‐of‐squares *F* test in GraphPad Prism Version 10.

## 3 Results

For all parameters tested, there were no significant effects of years of training or training hours/week.

### 3.1 Participant Characteristics

**FIGURE 1:**
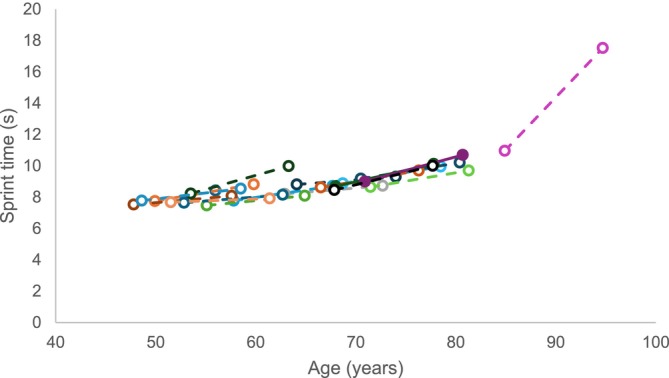
60‐m sprint time at baseline and 10 years later. Each colour and line indicate one individual.

The youngest participant was 48 and the oldest participant was 85 years at baseline in 2002. The athletes lost 1.1 cm of height over 10 years (*p* < 0.001. d = 0.857), but there were no significant changes in body mass or body mass index (BMI) (Table 1). Age graded performance did not change significantly after 10 years (Table 1), but 60‐m sprint time increased after 10 years (*p* < 0.001, d = 0.915), with stepwise regression demonstrating that changes in sprint performance were related to age (*R* ^2^_adj_ = 0.431; *p* = 0.004). It appeared that there was an accelerated increase in time required to complete a 60‐m sprint with age, such that the 85‐year‐old participant demonstrated a 6.6 s increase (Figure 1).

**TABLE 1:** Participant characteristics and training information (all *n* = 24 except age graded performance, *n* = 18).

	Baseline (2002)	10Y (2012)	*p*
Age (years)	61.9 ± 10.4	71.7 ± 10.4	—
Height (m)	1.74 ± 0.06	1.73 ± 0.06	**< 0.001**
Bodyweight (kg)	73.0 ± 7.9	73.0 ± 9.0	0.905
BMI	24.1 ± 1.7	24.3 ± 2.0	0.201
Training experience (years)	32.6 ± 20.2	42.6 ± 20.2	—
Training duration per week (hours)	7.10 ± 3.39	4.67 ± 2.42	**< 0.001**
Age graded performance (%)	93.7 ± 3.2	91.7 ± 5.5	0.100

*Note:* Values in bold indicate *p* < 0.05.

### 3.2 Whole Muscle Function and Morphology

**FIGURE 2:**
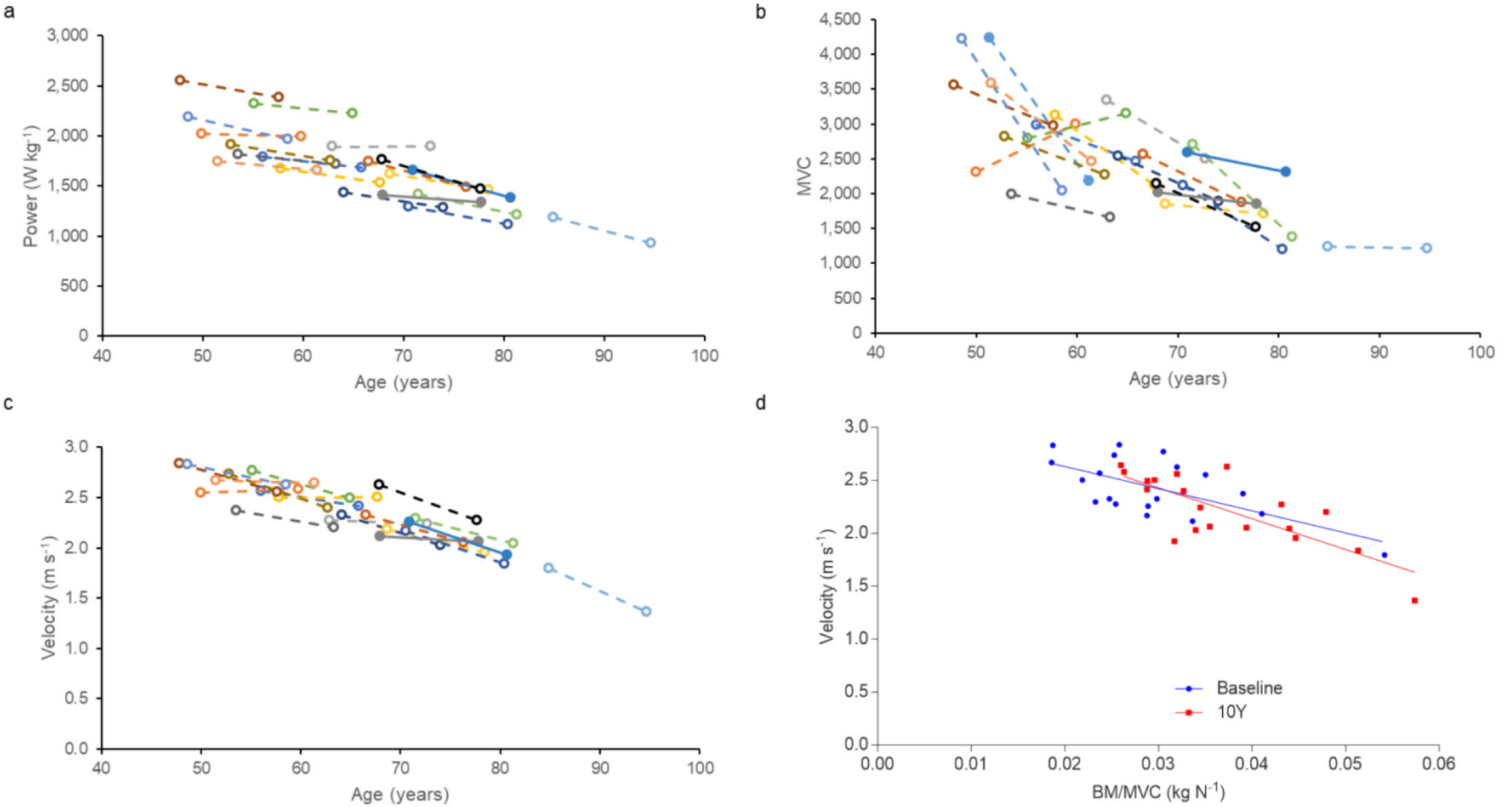
Whole muscle function measures at baseline and 10 years later (10Y). For (a–c), each colour and line indicate one individual. (a) jump power, (b) maximum voluntary contraction of knee extension (MVC) and (c) jump velocity. (d) Velocity versus body mass/MVC (BM/MVC) at baseline and at 10Y.

Power demonstrated in a countermovement jump decreased over time (*p* < 0.001, d = 1.893 Figure 2a), with greater reductions in power occurring in older participants (*p* < 0.001). Knee extension MVC was lower than baseline at the 10‐year time point (*p* < 0.001, d = 1.013; Figure 2b), irrespective of age at baseline. Countermovement jump velocity decreased over time in individuals (*p* < 0.001, d = 1.546) with greater reductions in older participants (*p* = 0.003; Figure 2c). Figure 2d demonstrates that velocity was inversely correlated with body mass/MVC at baseline (*R* ^2^ = 0.414, *p* = 0.003) and 10Y (*R* ^2^ = 0.605, *p* < 0.001), with no significant differences between slopes. As slowing of the muscle would result in a lower countermovement jump velocity at a given BM/MVC, the lower force generating capacity and not slowing of the muscle, is the main cause of the lower power after 10 years.

According to ultrasound measurement, neither VL thickness (*p* = 0.435) nor VL thickness/FCSA (rough indicator of fibre number) (*p* = 0.904) changed significantly over time in the athletes studied (Table 2). However, MVC/VL thickness did change over time (*p* < 0.001, d = 0.902; Table 2), irrespective of participant age.

**TABLE 2:** Vastus lateralis (VL) related measures; VL thickness (*n* = 23), maximum voluntary contraction force per muscle thickness of knee extension (MVC)/VL thickness (*n* = 20) and VL thickness/fibre cross sectional area (FCSA) (*n* = 23).

	Baseline (2002)	10Y (2012)	*p*
VL thickness (cm)	2.13 ± 0.35	2.16 ± 0.42	0.435
MVC/VL thickness (N cm^−1^)	820 ± 352	572 ± 270	**< 0.001**
VL thickness/FCSA (cm μm^−2^)	79 686 ± 29 220	79 935 ± 30 458	0.904

*Note:* Values in bold indicate *p* < 0.05.

### 3.3 Global Muscle Capillarisation

**FIGURE 3:**
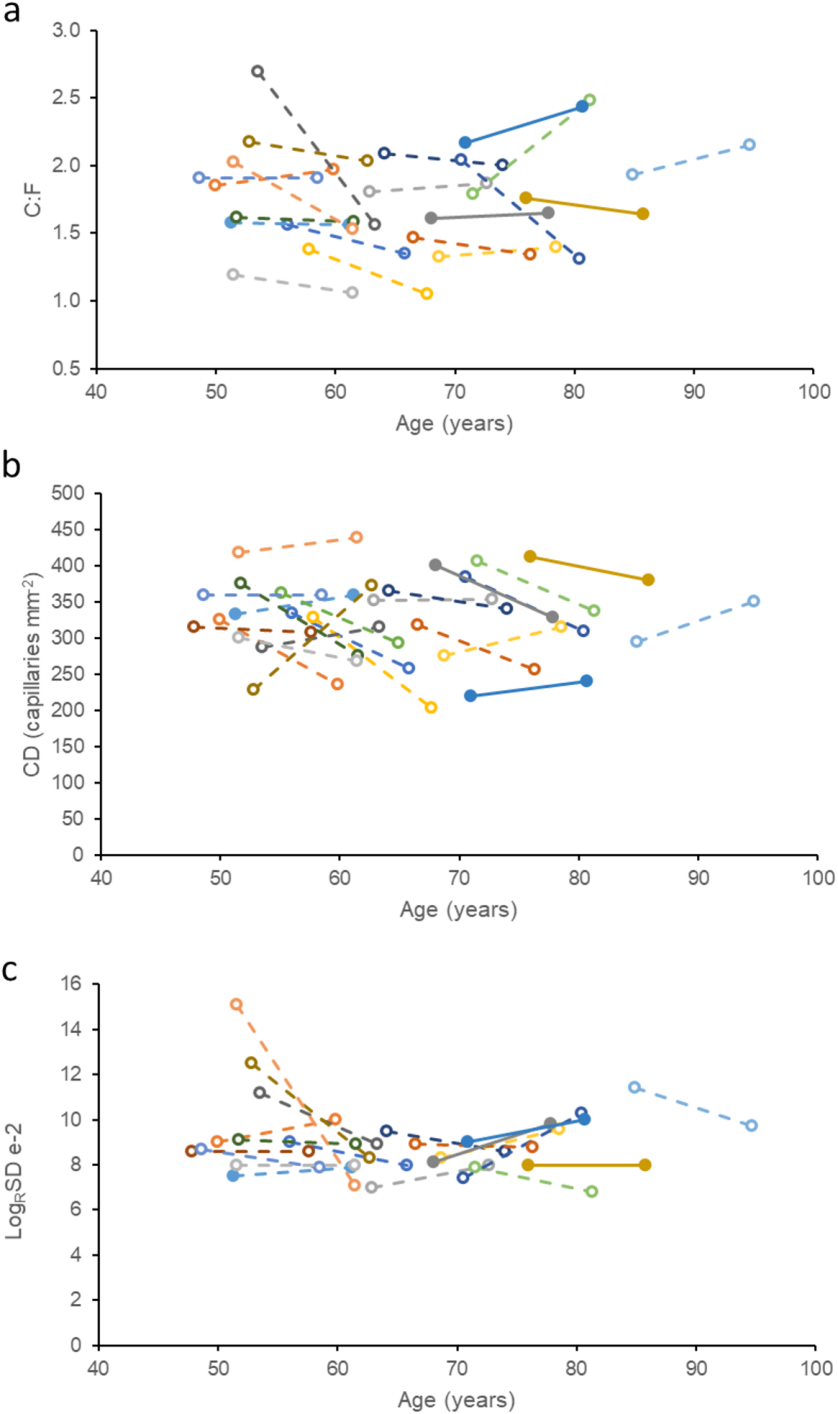
Capillary indices in masters sprinters at baseline and 10 years later. Each colour and line indicate one individual. (a) Capillary‐to‐fibre ratio (C:F), (b) capillary density and (c) LogRSD (a measure of capillary spacing heterogeneity).

There were no significant differences in global muscle capillarisation after 10 years; neither the C:F (*p* = 0.252; Figure 3a) nor the CD (*p* = 0.107; Figure 3b) changed significantly over the 10‐year period. The index of capillary spacing heterogeneity, Log_R_SD, also did not change significantly over time (*p* = 0.349; Figure 3c).

### 3.4 FCSA, Fibre Type Composition and Fibre‐Specific Capillarisation

**FIGURE 4:**
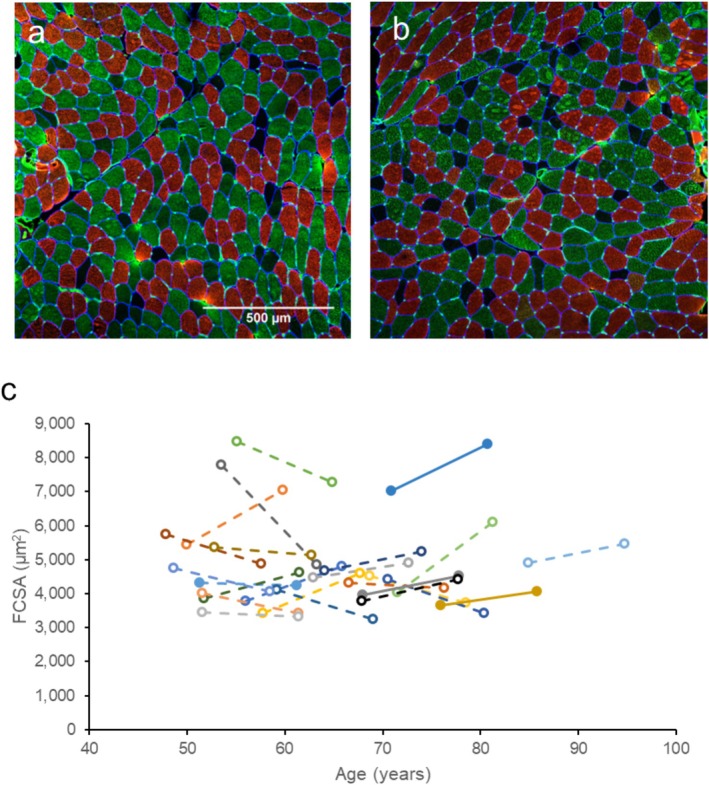
Histology of muscle biopsies. (a,b) Vastus lateralis muscle biopsy sections stained for Type I (red), Type IIa (green), Type IIx (unstained) and capillaries (green dots). (c) Mean fibre cross sectional area changes in masters sprinters at baseline and 10 years later. Each colour and line indicate one individual.

Average FCSA did not change significantly over 10 years (*p* = 0.585; Figure 4c). On a fibre type specific level, there were also no significant changes in FCSA of Type I, IIa and IIx fibres (Table 2). Fibre type proportions also did not differ after 10 years in fibre type I, IIa or IIx (Table 3).

**TABLE 3:** Fibre type specific measures. Fibre cross sectional area (FCSA), numerical fibre type proportion, local capillary to fibre ratio (LCFR) and capillary‐fibre density (CFD) (*n* = 24).

	Baseline (2002)	10Y (2012)	*p*
FCSA (μm^2^)			
Type I	4661 ± 1221	4791 ± 948	0.792
Type IIa	5094 ± 1838	5040 ± 1708	0.849
Type IIx	3751 ± 1195	3644 ± 1461	0.940
Fibre type proportion (%)			
Type I	44.2 ± 13.6	42.9 ± 14.1	0.543
Type IIa	45.8 ± 10.4	44.7 ± 14.0	0.252
Type IIx	10.0 ± 8.2	11.9 ± 11.1	0.382
LCFR			
Type I	1.69 ± 0.43	1.60 ± 0.44	0.349
Type IIa	1.68 ± 0.51	1.43 ± 0.40	**0.002**
Type IIx	1.20 ± 0.50	0.86 ± 0.36	**0.008**
CFD			
Type I	370 ± 58	335 ± 59	**0.022**
Type IIa	336 ± 51	304 ± 55	**0.034**
Type IIx	325 ± 52	295 ± 74	0.265

*Note:* Values in bold indicate *p* < 0.05.

A fibre specific reduction in LCFR occurred for Type IIa and IIx fibres after 10 years (*p* = 0.002, 0.008, d = 0.631 and 0.643, respectively) and CFD decreased for Type I and IIa fibres (*p* = 0.022and 0.034, d = 0.529 and 0.484, respectively; Table 3).

## 4 Discussion

The major finding from this study is that sprint and jump performance decreased in masters sprint athletes over 10 years with greater reductions in the older participants. These reductions were not explicable by self‐reported years of training nor training volume. The reduced performance was attributable to a decline in force generating capacity but not slowing of the muscle, which corresponds with the unaltered fibre type composition. However, the lower force generating capacity was not accompanied by a reduction in muscle size, FCSA or muscle capillarisation and thus suggests that other factors than muscle fibre morphology contribute to the decreased sprint and jump performance in sprint athletes over 10 years.

### 4.1 Muscle Performance

As mentioned in previous studies of fibre type grouping [23] and bone [18], significant increases in 60‐m sprint time occurred over 10 years in these athletes (fewer participants were included in this study due to tissue availability). The increase in time required to complete a 60‐m sprint was larger in older athletes and the accelerated decrement in 60‐m sprint performance mimicked the cross‐sectional data of masters 100‐m world records [2] and performance in other track and field events [1]. This supports the use of cross‐sectional data to study changes in athletic performance with ageing.

**FIGURE 5:**
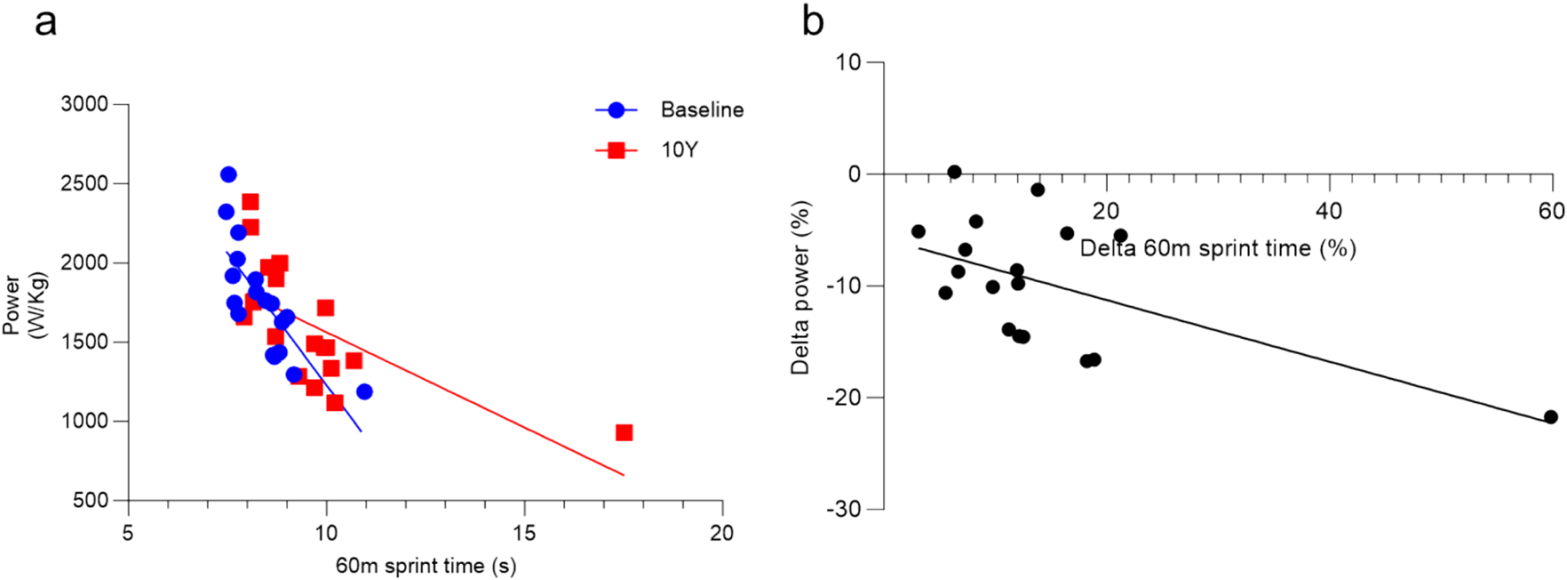
Sprint performance and jump power in masters sprinters. (a) 60‐m sprint time and jump power in individuals at baseline (blue, *R* ^2^ = 0.616; *p* = 0.0001) and at the 10 year follow‐up (red, *R* ^2^ = 0.444; *p* = 0.0026). (b) Percentage change in sprint time from baseline to 10‐year follow‐up (delta 60‐m sprint time) versus percentage change in jump power (delta power) (*R* ^2^ = 0.349; *p* = 0.0099).

The greater rate of power loss in older people, also seen in another 10‐year longitudinal study of people aged 20–90 [9], likely contributes to the larger decrements in sprint performance in older than younger athletes. This is supported by our observation that not only was there a good correlation between jumping power and 60‐m sprint performance (Figure 5a), both at baseline (*R* ^2^ = 0.616, *p* = 0.0001) and at 10‐year follow ‐up (*R* ^2^ = 0.444, *p* = 0.0026), but also between the decline in jumping power and 60‐m sprint performance (Figure 5b; *R* ^2^ = 0.349, *p* = 0.01). Here, we demonstrated that the reduction in sprint performance and jump power is accompanied by both a lower jump velocity and MVC over the 10‐year period. At face value, one could thus argue that the reduced power is the result of both a lower force generating capacity and slower contractile properties. However, because of the lower force generating capacity, the muscle will have to work at a slower part of the force–velocity relationship, and this, independent of slower contractile properties, could already result in a lower jumping velocity and 60‐m sprint performance [30]. To take this possibility into account, we also plotted the jump velocity as a function of BM/MVC and found no change in this relationship over the 10 years. This thus indicates that the slower jump velocity is attributable to a lower force generating capacity of the muscle, forcing the muscle to work at a slower part of the force–velocity relationship, and not a slowing of the contractile properties of the muscle tissue, corresponding with an unaltered fibre type composition over the 10‐year period.

A similar pattern in loss of power and velocity has been seen before in ageing, both cross‐sectionally [36] and longitudinally over 5 years [37], albeit in healthy older people, not master athletes. A subsequent study using unloaded and loaded countermovement jumps in young and older men and women revealed that the ageing‐related loss of power and velocity was explicable, as in the present study, by a lower force generating capacity rather than slowing of the muscle [31]. It is noticeable, however, that in the present study, the oldest participant demonstrated no reduction in MVC, and the reduced jump velocity must in that case have been attributable to a slowing of the muscle. In this context, it is interesting that the reduction in peak power demonstrated with a leg press was accompanied with a larger amount of muscle fibre slowing in mobility‐limited than that seen in healthy older people [7], and we therefore suggest that further studies may reveal that the ageing‐related decrement in power shifts from primarily being a consequence of weakening to slowing of the muscle in athletes and the normal population alike.

In contrast to the accelerated loss of power, the reduction in strength (MVC) over 10 years did not accelerate with ageing. This comports with a longitudinal study of young, middle aged and older people in which muscle maximal torque also appeared to decline (in absolute terms) similarly in all age groups [38]. An obvious explanation for a reduced force generating capacity would be a loss of muscle mass, but there was no significant reduction in VL thickness over the 10‐year study period in our athletes. What we did observe, however, was a reduced MVC/thickness, a rough indicator that the force generating capacity of the muscle tissue is diminished. Such a situation may occur when there is a reduced neuromuscular activation and/or a slowed rate of activation, as seen in healthy older people [6, 39], and a lower motor unit firing rate as seen in physically active older men [40, S1] and masters athletes [S2] compared to younger men. Although we show that VL thickness/FCSA did not change, indicating that the composition of muscle is maintained, it is possible that intermuscular adipose tissue increased after 10 years as direct measures were not taken. Increased fat infiltration occurs in ageing and is associated with impaired muscle strength [S3]. This may play a role in the reduction in MVC/VL thickness over the 10‐year period, although this is unlikely as it has been shown that resistance training prevents fat infiltration in muscle in older people [S4].

Whatever the cause of the decline in force generating capacity over the 10 years, it indicates that the training performed by these athletes did not prevent loss of muscle strength, something also seen over 10 years in older people who participated in strength training [S5]. Although one might argue that this loss of muscle strength is at odds with the known benefits of strength training to enhance the force generating capacity of a muscle, even in older adults [S6], it should be noted that power athletes were at any age better off than non‐athletes, and despite a larger rate of loss of power in absolute terms [S7], they will slip below the ‘disability threshold’ later in life [S8] as illustrated by Degens [S9]. Although the self‐reported—and hence not controlled—weekly training duration was reduced in the present study, our findings and those of Hughes et al. [S10] indicate that continued strength training cannot prevent the age‐related loss of strength the benefit is that sprint athletes will most likely have an increased number of quality‐of‐life years compared to non‐athletes.

### 4.2 Fibre Type Composition and Fibre Size

It is well recognised that declines in muscle mass occur with ageing, due to a loss of fibres [3] and a reduction in Type II FCSA [4, S11]. Here, we demonstrate that VL thickness did not change over the 10 years in the masters sprint athletes, and correspondingly, there FCSA was also maintained, indicating that there was neither fibre atrophy nor fibre loss. Although another study showed a decrease in quadriceps thickness in physically active older people over a 4‐year period, this decrease was less in people with greater physical activity [10]. Therefore, it may be that the type of training performed by sprinters (sprints alongside plyometrics and strength training) prevented a reduction in muscle thickness, which is not achievable by non‐specific physical activity alone. Interestingly, whereas long distance running is thought to be a less effective stimulus for growth or maintenance of muscle mass than strength [S5] or sprint training [S12], also long‐distance runners also maintain muscle FCSA over long periods (20 years) [17]. It should be noted, however, that the baseline age of long‐distance runners was similar to the youngest sprinters in the present study. Although Lazarus et al. [12] argue that meeting a certain threshold of physical activity is sufficient to maintain muscle mass with age, sprint training exercises may confer other benefits to bone density and strength compared to endurance running [18, S13, S14].

### 4.3 Muscle Capillarisation

Global measures of muscle capillarisation did not show a significant change over the 10‐year period in our sprint athletes, which corresponds with another study that showed no change in muscle capillarisation in 73‐ to 83‐year‐old men over a period of 7 years, despite fibre atrophy [S15]. Another study did, however, observe a 20.3% reduction in C:F over 12 years in sedentary older men without significant fibre atrophy [8]. Previous studies have found a close link between capillarisation and fibre size in youth and in ageing [S16], and the absence of any significant change in FCSA may thus explain the absence of significant changes in capillarisation in our study.

At the level of individual fibres, capillarisation changed somewhat, such as a reduction in the LCFR for Type II fibres after 10 years, indicating that there were fewer capillaries supplying those fibres, and a lower CFD for Type I and IIx fibres even though the size of these fibres was maintained, suggesting that there might be developing a slight disconnect between the size and capillary supply to a fibre in our sprint athletes during ageing. This indication for local remodelling of the vascular bed did, however, not result in an increased heterogeneity of capillary spacing, which is significant as an increased heterogeneity of capillary spacing is associated with impaired tissue oxygenation [S17, S18]. A clue as to the cause of a reduction in fibre type‐specific capillarisation may be the type of training performed by these athletes; although long distance running is associated with improved capillarisation, high‐intensity sprint interval training has been reported to potentially lead to reductions in CD and C:F without increases in fibre size [S19, S20], analogous to the situation here.

### 4.4 Longitudinal Versus Cross‐Sectional Declines in Performance

Here, we show that the longitudinal decline in sprint performance over 10 years mirrors that seen in cross‐sectional studies [2]. Even though sprint performance and the athlete's capacity to adapt to training are dependent on genetic traits [27, S21], our observation that the similar decrements in performance within individuals of a given age suggests that at a population level, deterioration of performance is independent of genetic variability and, according to our analysis, years of training and weekly training volume, and hence, ageing appears uniform rather than ‘individualistic’ [S22], something also concluded previously for athletes of all sorts of disciplines [1]. However, we also demonstrate that sprint training is beneficial in the maintenance of muscle mass, at both single cell and whole muscle levels over a 10‐year period. Perhaps most striking is the observation that the decline in muscle power and sprint performance is not related to any significant changes in muscle morphology, nor slowing of the muscle, but rather a decline of the force generating capacity of muscle tissue. What underlies this reduced force generating capacity is something for further investigation.

## 5 Limitations

Although the sample size is small compared to longitudinal studies of performance, it is relatively large given the highly trained nature of the population, the timespan studied and the age of the participants. Additionally, although training intensity and volume may have varied over the 10‐year period and are dependent on self‐reporting, we think this potential short‐coming is a minor issue as age graded performance did not change significantly over the 10‐year period. Finally, longitudinal studies are limited by a potential attrition bias, for example, those who best maintain their performance or are not subject to significant injury are more likely to return for follow‐up testing, but again, this at best plays a minor role, as no significant difference in athletic performance at 80 was found between those that continued and those that had stopped competing at 85 years [1]. Due to our sample size, we were unable to perform a sub‐analysis by age group, thus limiting insights into age‐related variations in muscle function and performance decline. Future studies focussing exclusively on older adults are needed to better understand muscle function and performance changes in this population.

## 6 Conclusion

Running performance in masters sprinters did decline over a 10‐year period, which was related to a lower force generating capacity, rather than slowing of the muscle. This reduction in force generating capacity and running performance was not accompanied by decrements in fibre size, fibre type composition or muscle capillarisation, and it remains to be seen what underlies this reduced force generating capacity.

## Ethics Statement

The study complied with the Ethical guidelines for authorship and publishing set by the *Journal of Cachexia, Sarcopenia and Muscle*.

## Conflicts of Interest

The authors declare no conflicts of interest.

## Supporting information


**Data S1** Supporting Information.
