# Supplementary material for: A 10‐Year Longitudinal Study of Muscle Morphology and Performance in Masters Sprinters
Source: J Cachexia Sarcopenia Muscle. 2025 Apr 27;16(3):e13822. doi: 10.1002/jcsm.13822 (PMC12034575; doi:10.1002/jcsm.13822)
Supplement: Supplementary file 1 — Data S1 Supporting Information. [file JCSM-16-e13822-s001.docx]

S1. Piasecki M, Ireland A, Jones DA, McPhee JS. Age-dependent motor unit remodelling in human limb muscles. Biogerontology. 2016;17:485-96. doi:10.1007/s10522-015-9627-3

S2. Piasecki M, Ireland A, Coulson J, Stashuk DW, Hamilton-Wright A, Swiecicka A, et al. Motor unit number estimates and neuromuscular transmission in the tibialis anterior of master athletes: evidence that athletic older people are not spared from age-related motor unit remodeling. Physiological reports. 2016;4:doi:10.14814/phy2.12987

S3. Goodpaster BH, Carlson CL, Visser M, Kelley DE, Scherzinger A, Harris TB, et al. Attenuation of skeletal muscle and strength in the elderly: The Health ABC Study. J Appl Physiol (1985). 2001;90:2157-65. doi:10.1152/jappl.2001.90.6.2157

S4. Goodpaster BH, Chomentowski P, Ward BK, Rossi A, Glynn NW, Delmonico MJ, et al. Effects of physical activity on strength and skeletal muscle fat infiltration in older adults: a randomized controlled trial. J Appl Physiol (1985). 2008;105:1498-503. doi:10.1152/japplphysiol.90425.2008

S5. Hughes DC, Ellefsen S, Baar K. Adaptations to Endurance and Strength Training. Cold Spring Harb Perspect Med. 2018;8:doi:10.1101/cshperspect.a029769

S6. Aagaard P, Suetta C, Caserotti P, Magnusson SP, Kjaer M. Role of the nervous system in sarcopenia and muscle atrophy with aging: strength training as a countermeasure. Scandinavian journal of medicine & science in sports. 2010;20:49-64. doi:10.1111/j.1600-0838.2009.01084.x

S7. Pearson SJ, Young A, Macaluso A, Devito G, Nimmo MA, Cobbold M, et al. Muscle function in elite master weightlifters. Medicine and science in sports and exercise. 2002;34:1199-206. doi:10.1097/00005768-200207000-00023

S8. Mithal A, Bonjour JP, Boonen S, Burckhardt P, Degens H, El Hajj Fuleihan G, et al. Impact of nutrition on muscle mass, strength, and performance in older adults. Osteoporos Int. 2013;24:1555-66. doi:10.1007/s00198-012-2236-y

S9. Degens H. Determinants of skeletal muscle hypertrophy and the attenuated hypertrophic response at old age. Journal of Sports Medicine & Doping Studies. 2012;1:doi:10.4172/2161-0673.S1-003

S10. Hughes VA, Frontera WR, Wood M, Evans WJ, Dallal GE, Roubenoff R, et al. Longitudinal muscle strength changes in older adults: influence of muscle mass, physical activity, and health. The journals of gerontology Series A, Biological sciences and medical sciences. 2001;56:B209-17. doi:10.1093/gerona/56.5.b209

S11. Lexell J. Human aging, muscle mass, and fiber type composition. The journals of gerontology Series A, Biological sciences and medical sciences. 1995;50 Spec No:11-6.

S12. Nuell S, Illera-Domínguez VR, Carmona G, Alomar X, Padullés JM, Lloret M, et al. Hypertrophic muscle changes and sprint performance enhancement during a sprint-based training macrocycle in national-level sprinters. Eur J Sport Sci. 2020;20:793-802. doi:10.1080/17461391.2019.1668063

S13. Piasecki J, McPhee JS, Hannam K, Deere KC, Elhakeem A, Piasecki M, et al. Hip and spine bone mineral density are greater in master sprinters, but not endurance runners compared with non-athletic controls. Arch Osteoporos. 2018;13:72. doi:10.1007/s11657-018-0486-9

S14. Korhonen MT, Heinonen A, Siekkinen J, Isolehto J, Alén M, Kiviranta I, et al. Bone density, structure and strength, and their determinants in aging sprint athletes. Medicine and science in sports and exercise. 2012;44:2340-9. doi:10.1249/MSS.0b013e318267c954

S15. Aniansson A, Hedberg M, Henning GB, Grimby G. Muscle morphology, enzymatic activity, and muscle strength in elderly men: a follow-up study. Muscle & nerve. 1986;9:585-91. doi:10.1002/mus.880090702

S16. Barnouin Y, McPhee JS, Butler-Browne G, Bosutti A, De Vito G, Jones DA, et al. Coupling between skeletal muscle fiber size and capillarization is maintained during healthy aging. J Cachexia Sarcopenia Muscle. 2017;8:647-59. doi:10.1002/jcsm.12194

S17. Piiper J, Scheid P. Diffusion limitation of O2 supply to tissue in homogeneous and heterogeneous models. Respiration physiology. 1991;85:127-36.

S18. Degens H, Deveci D, Botto-van Bemden A, Hoofd LJ, Egginton S. Maintenance of heterogeneity of capillary spacing is essential for adequate oxygenation in the soleus muscle of the growing rat. Microcirculation. 2006;13:467-76. doi:10.1080/10739680600776286

S19. Nyberg M, Fiorenza M, Lund A, Christensen M, Rømer T, Piil P, et al. Adaptations to Speed Endurance Training in Highly Trained Soccer Players. Medicine and science in sports and exercise. 2016;48:1355-64. doi:10.1249/mss.0000000000000900

S20. Hellsten Y, Gliemann L. Peripheral limitations for performance: Muscle capillarization. Scandinavian journal of medicine & science in sports. 2024;34:e14442. doi:10.1111/sms.14442

S21. Haugen TA, Solberg PA, Foster C, Morán-Navarro R, Breitschädel F, Hopkins WG. Peak Age and Performance Progression in World-Class Track-and-Field Athletes. Int J Sports Physiol Perform. 2018;13:1122-9. doi:10.1123/ijspp.2017-0682

S22. Degens H, Onambélé-Pearson GL. A simple framework to distinguish ‘individualistic’ from a ‘uniform rate’ of ageing within or between study populations. GeroScience. 2024;46:2765-9. doi:10.1007/s11357-023-00866-7
